# Cardiovascular Health Effects of Shift Work with Long Working Hours and Night Shifts: Study Protocol for a Three-Year Prospective Follow-Up Study on Industrial Workers

**DOI:** 10.3390/ijerph17020589

**Published:** 2020-01-16

**Authors:** Lars-Kristian Lunde, Øivind Skare, Asgeir Mamen, Per Anton Sirnes, Hans C. D. Aass, Reidun Øvstebø, Elisabeth Goffeng, Dagfinn Matre, Pia Nielsen, Hanne Siri Amdahl Heglum, Stine Eriksen Hammer, Marit Skogstad

**Affiliations:** 1 Department for Work Psychology and Physiology, National Institute of Occupational Health, Box 5330 Majorstuen, 0304 Oslo, Norway; Lars-Kristian.Lunde@stami.no (L.-K.L.); dagfinn.matre@stami.no (D.M.); 2 Department Occupational Medicine and Epidemiology, National Institute of Occupational Health, Box 5330 Majorstuen, 0304 Oslo, Norway; Oivind.Skare@stami.no (Ø.S.); Elisabeth.Goffeng@stami.no (E.G.); 3 Kristiania University College, School of Health Sciences, Box 1190 Sentrum, 0107 Oslo, Norway; asgeir.mamen@kristiania.no; 4 Østlandske Hjertesenter, Lilleeng Helsepark, Lillengvn 8, 1523 Moss, Norway; pas@cardio.no; 5 The Blood Cell Research Group, Section of Research, Department of Medical Biochemistry, Oslo University Hospital, Ullevål, 0450 Oslo, Norway; h.c.aass@medisin.uio.no (H.C.D.A.); reidun.ovstebo@medisin.uio.no (R.Ø.); 6 Ringvoll Occupational Health Service, Lilleeng veien 8, 1523 Moss, Norway; pia.nielsen@ringvollbht.no; 7 SINTEF, Department of Neuromedicine and Movement Science, Norwegian University of Science and Technology, 7067 Trondheim, Norway; hanne.siri.heglum@novelda.no; 8 Novelda AS, Strandveien 43, 7067 Trondheim, Norway; 9 Department of Chemical and Biological Work Environment, National Institute of Occupational Health, Box 5330 Majorstuen, 0304 Oslo, Norway; Stine.Hammer@stami.no

**Keywords:** biomarkers of inflammation, cardiovascular disease, arterial stiffness, carotid intima-media thickness, disease prevention, shift work, occupational, health promotion, physical activity, sleep deprivation, work organization

## Abstract

There is a plausible association between shift work and cardiovascular disease (CVD), which may be due to disruption of the circadian rhythm causing hormonal changes and metabolic disturbances, resulting in high blood pressure, atherosclerosis, diabetes, and being overweight. However, few studies have investigated the association between several consecutive long work shifts, including night shifts, and risk factors for developing CVD. Moreover, knowledge is lacking on factors that may modify or enhance this suggested relationship. The study period is planned from the third quarter of 2018 to the fourth quarter of 2021, and will involve 125 industrial employees at two Norwegian enterprises producing insulation. The work schedule is either rotating shiftwork (morning, evening, night) or regular day work. At baseline, we will measure blood parameters, including markers of inflammation, lipids, and glycosylated hemoglobin. We will also collect measures of blood pressure, resting heart rate, arterial stiffness, carotid intima-media thickness, and aerobic fitness. At the end of baseline data collection, a subgroup will undergo a supervised high-intensity interval training intervention for eight weeks, initiated by the Occupational Health Service. At one-year follow-up, we repeat baseline measures with added measures of heart rate variability and additional five weeks monitoring of sleep and physical activity, and assessment of respirable dust. At the two year follow-up, we will measure CVD risk factors before and after a planned three-month shutdown in one of the studied plants. We will also assess respirable dust, monitor sleep, and compile a one-year retrospective detailed overview of working hours. A final data collection, similar to the one at baseline, will be carried out after three years. We will use a comprehensive set of methods to identify the effects of shift work with long working hours and night shifts on cardiovascular health. This will provide new knowledge on the association between early manifestations of CVD and occupational exposure to shift work. Further, we can study whether work organization such as extensive overtime, sleep loss, and dust exposure have detrimental effects, and if a three-month cease in shift work or increased physical activity will modify early manifestations of CVD.

## 1. Introduction

The work market and work organization of today is highly affected by economic globalization. Amongst other effects, a result of this is the use of nonstandard working hours, which may, in broad terms, refer to work outside regular daytime. A common term used for this type of work organization is shiftwork, which may be two-shift rotation (commonly day and evening work) or three-shift rotation (day, evening and night work). Another term is long working hours, often defined as more than 8 h per day, and/or more than 40 h per week [1,2]. Work schedules may be combinations of shiftwork and long working hours.

Nonstandard working hours is previously suggested to increase the risk of ill health, in terms of e.g., work accidents, anxiety, sleep deprivation, increased alcohol consumption, decreased immune function, stomach and bowel symptoms, cardiovascular disease (CVD), and musculoskeletal health issues [1,3].

In the Western world, cardiovascular disease is a major cause of early death, and it is stated that 10–20 percent of all deaths caused by cardiovascular diseases in persons of working age are related to their work characteristics [4]. A study published in the late 1950s was one of the first to point out a possible association between occupational stress measured as working hours and hospitalizations due to heart attack [5]. In a systematic review with a meta-analysis including over two million subjects published in 2012, researchers found associations between shift work and several types of vascular events [6]. Another recent systematic review including a meta-analysis on 173,000 individuals similarly reflected an increased risk of cardiovascular disease for shift/night workers compared to day workers [7]. From analyses on their material, they estimated an increased risk of approximately seven percent for every five years of shift/night work after the initial five years. Another systematic review with a meta-analysis, determined, based on prospective observational studies, that there was approximately 40 percent excess risk of coronary heart disease for individuals undertaking long working hours [8].

There are several plausible mechanisms for why shift work with long working hours and night shifts may cause CVD. One suggested factor is the prolonged exposure to psychological stress, leading to hypersecretion of cortisol and catecholamines, which may contribute to disorders increasing risk for CVD [9,10]. Decreased secretion of melatonin due to “light at night” has also been suggested to cause metabolic disturbances [11,12]. Shift work can disturb sleeping patterns, and thereby affect insulin resistance and the immune system with the result of inflammation. These phenomena may increase the risk of diabetes and cardiovascular diseases [12,13].

Furthermore, physical inactivity in leisure, unhealthy diet, increased alcohol consumption, being overweight, smoking, likelihood of ignoring symptoms of disease, elevated blood pressure, and type 2 diabetes are all mechanisms that have been suggested to be involved in the association between shiftwork and CVD [8,14].

Since shift work has been associated with several risk factors and outcomes of CVD [6,7,15,16,17,18], it is also possible that the relationship between long working hours and coronary heart disease is moderated by shift work and vice versa. However, in their meta-analysis, Virtanen and colleagues restricted their analyses to day workers with long working hours only, and found that the association was still valid [8]. This does not rule out the possibility of an even higher risk for workers with the combination of both shift work and long working hours.

In published reviews, there is commonly a lack of information on exposure, in terms of shift organization, duration of shifts, overtime etc. and/or the level of quality on cardiovascular outcomes [6,7,14,15,19]. Furthermore, the outcome of shift work and lack of sleep could be similar [20]. The self-reporting of sleep duration or insomnia instead of objective measurements, along with self-reporting of outcomes like hypertension and diabetes may introduce biases and misclassification when aiming to untangle the relationship between shift work, including long working hours/night shifts and cardiovascular health [21,22].

To the author’s knowledge, very few studies have investigated the relationship between several consecutive shifts including long working hours and night work, and CVD risk factors, neither during the shift nor across a significant follow-up period. Additionally, evidence is lacking on possible mediating factors like physical inactivity, smoking, and obesity [7,23]. In addition, one must bear in mind that industrial workers are at risk of exposure to respirable dust which is known to affect the inflammatory process, leading to an increased risk of CVD [24]. The implementation of workplace health promotion initiatives are also sought for, with the aim of limiting CVD risk in shift workers [7].

With this study, we will use a comprehensive set of methods including novel technology to identify the physiological effects of shift work on the cardiovascular system. To do this, we will study a set of blood parameters, including a selected set of biomarkers for inflammation, lipids, and glycosylated hemoglobin. Further, we will carry out measurements of maximal oxygen consumption, resting heart rate, heart rate variability (HRV), blood pressure, measures of arterial stiffness, and ultrasound examination of the carotid artery, including carotis intima-media thickness (cIMT). Additionally, we will include measures of sleep problems/sleepiness, physical activity (PA) levels, and respirable dust, and investigate the effects of an eight-week, high-intensity interval training (HIIT) intervention and a three-month shutdown of production.

### Research Objectives

1. The main objective is to determine, prospectively over a period of three years, whether several consecutive days/years with long working days included in a shift plan with night work are associated with unhealthy effects on the cardiovascular system.
2. The secondary objective is to evaluate the associations between shift work/long working hours, sleep patterns, and CVD-risk outcomes.
3. The third objective is to evaluate whether a three-month scheduled plant shutdown affects CVD-risk outcomes in workers.
4. The fourth objective is to evaluate if eight weeks of HIIT intervention affect the selected measures of cardiovascular health.

## 2. Materials and Methods

The study is registered with the ISRCTN registry (ISRCTN 42416837 (2018)).

### 2.1. Setting and Study Population

We will include two Norwegian enterprises (Plant A and Plant B) which are mainly involved in the production of insulation products. All employees (*n* = 213) will be informed of the research project via information meetings carried out by the Occupational Health Service in collaboration with plant representatives, and will be given the opportunity to participate. Based on previous experience, we expect that approximately 60% of the workers will accept the invitation. Baseline and follow-up measurements will be carried out over a period of three years in order to collect relevant data from the study participants. Overall, data collection consists of a questionnaire, a work hours register, aerobic capacity testing, and medical examinations with measurements of blood pressure, arterial stiffness, cIMT, and blood sampling. Additionally, we will include measures on sleep, physical activity, and respirable dust. The study has been approved by the Regional Ethics Committee in Oslo, REC South East B (Reference number: 2018/1258).

#### Shift Work Schedule and Work Content

The regular shift work schedule is a “five-shift plan”. At plant A, it consists of a five-week plan with seven night shifts, in which five are of eight h duration and two are of 12 h duration. Furthermore, the schedule includes five evening shifts lasting eight hours. As for day shifts, of which there are altogether seven, the duration is eight h on weekdays, but 12 h on weekends. The schedule for plant B is similar as to that of A when it comes to the night shift, but for plant B, only four evening shifts lasting 8 h are included. This group of workers have altogether seven day shifts, of which three are of 12 h duration and four are of 8 h duration. Both plants allow workers to take extra shifts and switch shifts between workers. All workers will have five week’s annual leave in addition to days off when changing from one type of shift to another (Table 1). Normally, workers will rotate between work tasks, consisting of supervising the production by watching computer screens, regular controls and maintenance of machines, quality control of products, and driving forklifts. For the nine months leading up to the shutdown in plant B, the work schedule will be intensified for employees at this plant as they are preparing for shutdown. Therefore, the shift work schedule will include 12-h shifts on all working days for these workers in this period, before they return to their normal shift plan after the shutdown (Table 2).

### 2.2. Exclusion Criteria for Participation

Subjects will be excluded from participation if diagnosed with severe heart or lung disease, cancer, or if they are showing very high blood pressure levels at the medical examination (>180/110). In subjects where unknown medical issues are discovered during examinations, the affected individual will be assigned to an appropriate medical institution or their general practitioner for further investigation.

### 2.3. Data Collection Period

**Figure 1:**
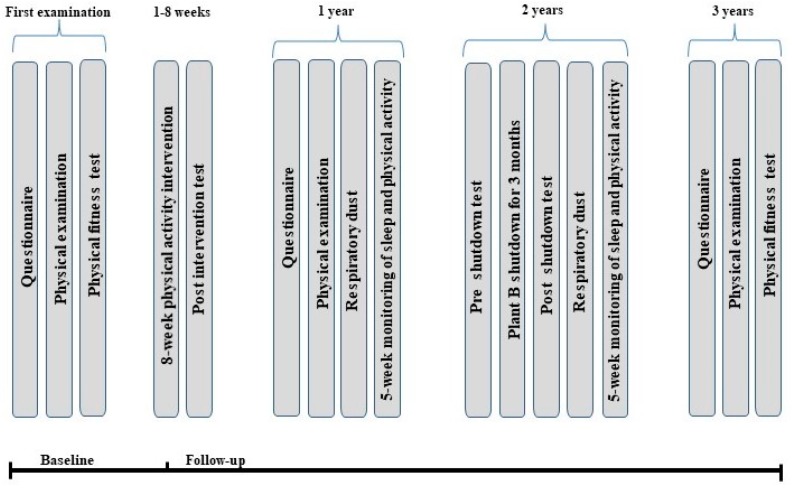
Time line for data collection.

Data collection is planned to start in the third quarter of 2018 and finish in the fourth quarter of 2021. All subjects will be followed for a total of three years after baseline measures, with data collection at baseline, after one year, and three years of follow-up. Furthermore, we will carry out three investigations in subgroups within the three-year study period. These investigations aim to target different important aspects of shift work and health, and will include: (1) postexaminations of subjects participating in an eight-week physical activity intervention initiated by the Occupational Health Service; (2) a five-week monitoring of sleep and physical activity of a subgroup at one-year follow-up; (3) a pre- and post-test of shift-workers, including monitoring of sleep and sleep parameters at plant B in connection to a planned three-month temporary shut-down of the plant two years after baseline. See Figure 1 for a detailed timeline of the full data collection period.

### 2.4. Data Collection Procedure at Baseline

#### 2.4.1. Questionnaire

At baseline, subjects will answer a self-administered questionnaire regarding education level, work experience, medication use and history, and lifestyle factors. This will include specific questions on diseases and illness, medicines, with additional focus on those affecting the cardiovascular system, e.g., blood pressure and cholesterol lowering medication, type and quantity of physical activity, smoking habits, type of shiftwork, work history, and previous shift work.

#### 2.4.2. Blood Collection and Plasma Preparation

We will collect glycosylated hemoglobin (HbA1c) in Ethylene diamine tetra acetic acid (EDTA) blood. Serum for the investigation of lipids (cholesterol, low-density lipoprotein (LDL), high-density lipoprotein (HDL), and C-reactive protein (CRP) will be collected on gel tubes and centrifuged at 35 × 1000 rpm for 15 min within 60 min after the blood has been drawn from a vein. The Department of Medical Biochemistry Oslo University Hospital will analyze the samples within 48 h. HbA1c EDTA blood will be analyzed with a Tosoh G7 HPLC analyzer (Tosoh Bioscience, Inc. San Francisco, CA, USA) using the “high performance liquid chromatography” separation principle. Cholesterol, LDL and HDL in serum will be analyzed by enzymatic colorimetric method in the Cobas 8000 (Cobas 8000 Modular Analyzer Roche Diagnostics). CRP will be assed in serum using the particle-enhanced immune turbidimetric method on Cobas 8000.

We will sample nonfasting blood (tubes containing K2EDTA or serum Sep Clot Activator) at the same time of the day on all three occasions for the investigation of lipids (cholesterol, LDL, HDL), HbA1c, and CRP. The serum tubes will be centrifuged at the workplace of the participants and the blood samples will be delivered to the Department of Medical Biochemistry, Oslo University Hospital, who will analyze the samples within 24 h. Cholesterol, LDL, and HDL in serum will be analyzed by an enzymatic colorimetric method in the Cobas 8000 c702. HbA1c in EDTA blood will be analyzed with a D100 TM BioRad, which uses high performance liquid chromatography as the separation principle. Furthermore, CRP in serum will be quantified by particle-enhanced immunoturbidimetric method with a Cobas 8000 (Cobas 8000 c702).

#### 2.4.3. Quantification of Plasma Cytokines

Whole blood will be collected in EDTA plasma tubes and inverted five times prior to centrifugation (2000× *g* 15 min). Plasma will be collected, mixed, and aliquoted into approximately 500 μL volumes in 1.5 mL Eppendorf tubes and stored at −80 °C until further analysis. All samples will be thawed on ice and centrifuged at 10,000× *g* for ten minutes prior to appropriate dilution. Plasma levels of Interleukin-6 (IL-6), Monocyte chemoattractant protein-1 (MCP-1), Tumor necrosis factor-alpha (TNF-*α*), P-Selectin, CD40 ligand (CD40L), and Leptin will be determined using a magnetic bead-based Luminex screening six-plex assay, and by a single plex Adiponectin assay. The bead assays will be processed and analyzed with a Luminex IS 200 instrument (Bio Rad, Hercules, CA, USA). An in-house longitudinal control will be used on each plate (*n* = 6) to determine the intra- and inter-assay percentage coefficient of variation.

#### 2.4.4. Blood Pressure and Resting Heart Rate

Following five minutes of seated rest, we will measure blood pressure and resting heart rate (RHR) from the subjects left arm using BpTRU^®^ (Bp TRU medical devices, Coquitlam, CO, Canada). For systolic (sBP) and diastolic blood pressure (dBP), the mean reading out of three measurements (for each measure) carried out in intervals of one minute will be used.

#### 2.4.5. Central Pulse Pressure and Arterial Stiffness

The arterial pulse waveform is the sum of the forward pressure wave and the backward propagating wave that is reflected from the periphery afterwards. We will measure central blood pressure, augmented pressure, and its index in a noninvasive manner using a SphygmoCor XCEL (Atcor Medical, New South Wales, Australia). A standard blood pressure cuff will be applied on the left arm on the radial artery to obtain pressure waveforms. The system produces a central aortic waveform from the pressure oscillations in the partially-inflated cuff [25]. In order to measure pulse wave velocity (PWV), we will place a blood pressure cuff on the right thigh approximately 200 mm from the inguinal area. We will measure the distance between the cuff over the femoral artery and the suprasternal notch, and from the suprasternal notch to the pulse of the carotid artery. Aortic PWV will be calculated from the distances between measurements points and the measured delay of time between 10 proximal and distal waveforms. Prior to the test, the participants are requested not to smoke, eat big meals, or consume alcohol, as recommended by the manufacturer [25].

#### 2.4.6. Carotid Intima-Media Thickness

Since atherosclerosis is associated both with an increased cIMT and the development of carotid plaques [26], we will use cIMT as a measure for subclinical atherosclerosis. cIMT is defined as the distance between the lumen-intima interface and the media-adventitia interface and plaque as a focal structure that protrudes into the arterial lumen of at least 0.5 mm or 50% of the surrounding cIMT value, or that demonstrates a thickness of 1.5 mm measured form the media–adventitia interface, as reported in the Mannheim consensus document [27]. Ultrasound scanning will be performed by a GE Vivid E9 system (GE Healthcare, Chicago, IL, USA) using a linear L9 array transducer. A minimum 10 mm length of both common carotid arteries at least 5 mm proximal to the carotid bifurcation will be scanned in an area free of plaques and with a clearly visible lumen–intima interface, using the semi-automated software of the Vivid system (GE Healthcare, Chicago, IL, USA). We will record the mean cIMT value of three end-diastolic images from both sides, as well as the maximal cIMT. In addition, the bifurcation and internal and external carotid arteries will be assessed for the presence of plaques. Data will be analyzed offline with the Echopac PC v12 (GE Healthcare, Chicago IL, USA). The total plaque burden of all eight carotid segments will be calculated as a score ranging from 0 to 24 as defined by ten Kate et al. [28].

#### 2.4.7. Aerobic Fitness

Aerobic fitness will be determined by maximal oxygen consumption (VO_2max_). We will test all subjects by a standardized graded test using a cycle ergometer (Monark 874 E, Monark Exercise AB, Vansbro, Sweden) while measuring oxygen uptake with a Cosmed K5 metabolism analyzer (Cosmed Srl, Rome, Italy). Starting with an initial load of 70 Watts and a cadence of 70 revolutions per minute (RPM), load will thereafter be increased by 35 Watts every minute. When the subjects fail to keep up with a cadence of minimum 60 RPM despite encouragement, they will be considered exhausted. We will use the 30 s averaged interval with the highest measurements to calculate participant’s VO_2max_.

### 2.5. Physical Activity Intervention

The PA intervention will be carried out by the local Occupational Health Service, with support from Kristiania University College, School of Health Science. The intervention group will consist of all volunteering shift workers in plant A (out of 51 eligible workers), while a control group of shift workers will be recruited from plant B (out of 55 eligible workers). Subjects in the intervention group will perform three supervised training sessions per week for eight weeks. Training sessions will be carried out on a work site in the enterprise’s own training facilities, and will be guided by a physical therapist. Based on participant’s own choice, sessions will be performed by cycle ergometer, row machine, steep hill walking, or running. Dedicated motivators will log all performed sessions during the eight-week intervention period, and will afterwards continue to motivate coworkers to undertake regular PA during the three-year follow-up. The motivators will have regular meetings with the project leaders throughout the follow-up period. The total duration of an interval session will be 17 min, including 10 min warm-up and 3 min cool-down at 60–70% maximal heart rate (HRmax) [29]. During warm-up, participants should not experience any shortness of breath, and ideally, should rate their perceived exertion to be 11–13 on a Borg scale [30]. In extension of the warm-up, the subject will carry out a single four-minute interval at 85–95% of HRmax, rating 16–19 on Borg scale. Subjects will be instructed to strive to attain the desirable HR range within the two first minutes of the four-minute effort. All subjects will be equipped with a personal heart rate measurement device that includes Personalized Activity Intelligence (PAI), in which a high level of PAI seems to prevent or postpone CVD [31,32].

### 2.6. Data Collection Procedure during Follow-Up

#### 2.6.1. Eight-Week Follow-Up

At eight weeks, data will be collected in a similar manner as for baseline, with the exception of cIMT measurement. All participants will additionally answer a self-administered questionnaire focusing on changes in medication (e.g., statins and antihypertensive medication), report their physical activity, and their present work tasks.

#### 2.6.2. One-Year Follow-Up

At the one-year follow-up, we will carry out a reexamination similar to the one at baseline excluding blood analyses, cIMT, and VO_2max_. In addition, we will measure HRV in a subgroup of 15 rotating shift workers and 15 former shift workers presently engaged in day work, during a cross-shift period of five weeks after one year of follow-up. This is a noninvasive procedure that takes approximately 20 min. We will use high definition ECG and beat-to-beat blood pressure measurements and the validated software in the Task Force Monitor^®^ (CNSystems, Graz, Austria). This will give indices of sympathetic and parasympatetic activity by the high frequency, low frequency, and very low frequency oscillations. We will also assess time domain-derived parameters [33]. Sleep will be measured subjectively by electronically-administered questionnaires [34] sent to the participants every evening at 21:00 during the five-week period. Objective measures of sleep will be actigraphy and radar monitoring. Each participant will wear an Axivity AX3 accelerometer (Axivity Ltd., Newcastle, UK) for two weeks, which, in addition to sleep, will measure the physical activity level for that period. For five weeks, each participant will also have a bedside XeThru x4m200 radar module capable of recording body movements and respiration rate during sleep [35]. A user button on the bedside module will let the participants input exact bedtime and risetime. Parameters of sleep including total sleep time, wake after sleep onset, sleep onset latency, number of awakenings, and sleep efficiency will be inferred from the recorded signals and the user inputs, and will be used to assess overall sleep patterns during the entire shift-period of five weeks.

At this point, we will additionally measure respirable dust. This assessment includes personal sampling using a JS holdings cyclone (FH022) and a custom-built pump with flow 2.2 L/min during a full day shift among six workers at both plants for three consecutive days. The flow rate will be recorded with a rotameter (Aalborg instruments, Denmark) pre- and post-sampling. The respirable cyclone is attached in the breathing zone of the worker. Particles will be collected on 37 mm polyvinyl filters with a 5 µm pore size (Millipore). All filters are weighed before and after exposure using a microbalance Sartorious MC 5 (Sartorius AG, Göttingen, Germany) after the filters have been acclimatized in a temperature- (20 ± 1 °C) and humidity- (40 ± 2%) controlled laboratory for a minimum of one day. Direct measure of particle size distribution and number concentration will be assessed using two particle counters, i.e., an aerodynamic particle sizer spectrometer (TSI 3321) and a scanning mobility particle sizer spectrometer (classifier model TSI 3082 and detector model TSI 3756). These instruments will be placed near the production line, i.e., in the area where the workers are present when they are handling the products manually.

#### 2.6.3. Two-Year Follow-Up (Plant B Shutdown)

After two years, there will be a three-month shutdown of Plant B due to maintenance, where its workers will be on paid leave. Directly prior to, and immediately after this shutdown, we will carry out examinations of the affected subjects. Measurements will be done in a similar manner as baseline, except from measures of cIMT and VO_2max_. At this time point, we will also monitor sleep quality and physical activity as in the one-year follow-up, and collect one-year retrospective detailed working hours from the employer’s register and do a repeated measure of respirable dust after the reopening of the plant.

#### 2.6.4. Three-Year Follow-Up

The final data collection, identical to the one at baseline, will be carried out three years after baseline. The questionnaire will be similar to the previous ones, but will, in addition, include information on any change in lifestyle habits, such as smoking and nutrition, for the previous year.

### 2.7. Data Management

#### Data Storage

All electronic files will be placed on an encrypted database protected by two-step verification and a firewall. All physical documentation will be stored in facilities specialized for this purpose, with a physical lock. No unauthorized personnel will have access to these files or facilities.

### 2.8. Statistical Analyses

The statistical analyses will address the four research objectives stated earlier.

To elucidate the first objective, we will study the effect of shift-work on CVD-risk outcomes during a three-year follow-up using measurements at baseline and at the one- and three-year follow-ups. The primary comparison will be between the three-year follow-up and baseline. The exposure variable will be dichotomous with categories “shift-worker” and “day-worker” (reference category).

For the second research objective, we plan to use objectively measured sleep and subjectively measured sleep quality during the five-week shift period as continuous exposures variables and investigate associations to CVD risk factors. We will also include an analysis where sleep is considered a mediator between shift-work and CVD outcomes.

The third research objective concerns the effect of a three-month scheduled plant shutdown on CVD-risk outcomes. Measurements will be done before and after the shutdown, and we aim to investigate possible changes in this period.

The fourth research objective studies the effect of an eight-week HIIT intervention. We will look at the change in CVD-risk outcomes for the intervention group, and also compare this to the corresponding change for the control group. In addition to this intention to treat type analysis, we plan also to do a per-protocol analysis, where only persons having attended at least 40 percent of the training sessions will be counted as part of an HIIT group. The remaining persons will be considered to be part of the control group. We will also measure their degree of physical activity during the eight-week follow-up using the PAI index, and we will investigate whether this measure is associated with changes in the CVD-risk outcomes.

All the research questions involve longitudinal data with two or more time points. Linear and logistic mixed models will be used for all research questions. For the continuous outcomes, a linear mixed model will be used to analyze the data with a random intercept for worker, and adjustment for age, gender, and smoking. For the dichotomous outcomes, a logistic mixed model will be used with the same random-effect and fixed-effect variables. To control for multiple testing, we will use *q*-values, which is the false discovery rate equivalent for *p*-values [36].

#### 2.8.1. Loss to Follow-Up, Compliance, and Missing Data

Based on previous experiences, we expect approximately 60% of the total workforce at the plants to volunteer for participation in the project. The project will be deeply anchored with the plant’s leadership. Participants will be closely followed by the Occupational Health Service after inclusion, and we expect 10–20% loss to follow-up for the whole 3-year period. For the intervention group, 80–90% compliance is expected for the planned and supervised sessions. Outcome will be censored in case of people leaving the employment at the plants due to unemployment, disability pension, or rehabilitate pension. Missing outcome values will be taken into account by the mixed model approach, and no prior imputation is necessary. For the adjustment variables, we do not expect much missingness.

#### 2.8.2. Study Power

Based on the assumption of 60% participation, we expect approximately 125 participants at baseline. Furthermore, our power calculation assumes a dropout of 10% after one year, and 20% after three years.

We consider the first research question as the primary one, and to evaluate statistical power, we focused on the effect of shift work on change in systolic blood pressure from baseline to last follow-up. We fixed the between-subject and within-subject variability to values estimated in a similar longitudinal study [37], i.e., we assumed a between-worker standard deviation of 9.3 and a within-worker standard deviation of 7.8. Furthermore, we assumed an effect size corresponding to a yearly increase of 1.5–2 units in blood pressure in the shift group compared with the reference group of day workers. Based on these assumptions, we obtained a power of 0.56–0.80 of getting a significant effect of shift work on change in systolic blood pressure. However, to better account for the fact that we have multiple outcomes, we took this power analysis one step further. We assumed that of the variables included in our study, 1/3 had an effect size of the same magnitude as for the systolic blood pressure, while for the other 2/3 of the outcomes, we assumed there were no effects of shift-work exposure. We adjusted the *p*-values for multiple-testing using a Bonferroni correction and obtained a power of 0.57–0.95 of getting at least one significant finding when adjusted for multiple testing.

## 3. Discussion

This study will be one of very few studies investigating relationships between shift work with long working hours and night work and early manifestations of cardiovascular disease in an occupational setting [38,39,40]. We will be doing this prospectively, using a comprehensive and partly novel set of methods. Furthermore, the three implemented investigations regarding plant shutdown, sleep monitoring, and PA-intervention will provide additional knowledge on important aspects of the investigated association between of shift work and cardiovascular health.

Systemic inflammation may contribute to the development of various manifestations of CVD [41]. The development and progression of atherosclerotic CVD is very complex, and has several different stages involving a range of inflammatory agents [42]. Abnormal levels of several pro- and anti-inflammatory agents have been linked to increased risk of developing CVD; these include CRP, MCP-1, TNF-*α*, Il-6, CD40L, and *p*-selectin [43,44,45]. Thus, these markers will provide information on our participant’s CVD risk and progression of risk during the follow-up. Further, we will investigate endothelial dysfunction in the arterial wall by measuring the stiffness of the arteries and through ultrasound of the cIMT. These are noninvasive methods used as proxies for atherosclerosis, and thus, predictors of future CVD. As for cIMT, in a normal, healthy male, one would expect the yearly progression of thickening to be approximately 0.012–0.015 mm [46]; an increase in cIMT of 0.1 mm would increase risk of myocardinal infarction by 10–15 percent and stroke by 13–18 percent [47]. Therefore, this measure provides highly relevant information regarding CVD development. To our knowledge, few research studies within occupational medicine have investigated cIMT. Fujishiro et al. found an increased cIMT thickness in manual workers with low job control [48], while others suggested that shift-work accelerated the atherosclerotic process and was associated with subclinical CVD [40,49]. A high central blood pressure, pulse pressure, augmented pressure, index, and PWV reflect arterial stiffness, i.e., s the load put on the left ventricle and coronary and cerebral vessels. This is a measure shown to have better predictability of cardiovascular outcomes than standard brachial measured blood pressure [50,51,52], and is also a predictor of various other organ disorders and mortality [53]. The recording of central blood pressure and PWV in this study will enable us to investigate possible differences in arterial stiffness between groups and its change during the follow-up.

### Strengths and Limitation

A major strength of this study will be the comprehensive set of health measures and biomarkers used. These measures will provide in-depth information on the participating individual’s cardiovascular health, and improve knowledge on the cardiovascular health effects of shift work with night work and long working hours. The comprehensive set of biomarkers are relevant to a wide range of globally-challenging diseases, and for several of these measures, there is no or limited knowledge on their relationship to shift work.

Another strength is the controlled environment in this study, providing a detailed description of the shift organization, work hours, and sleep status of an individual level. We additionally measure factors as respirable dust exposure and physical activity, both linked to CVD risk factors [54,55], in order to gain control over other aspects which are potentially important in CVD development. This will enable us to better study effects of shift work on early manifestation of CVD.

The three-month shutdown at plant B within our study period will provide us with the opportunity to investigate whether there are any detectable changes in parameters of cardiovascular health connected to this period where participants are not exposed to shift work. In addition, the eight-week HIIT intervention carried out by the Occupational Health Service will provide great information on the effects of PA on the parameters of cardiovascular health, and additionally, will allow us to investigate the mediating effect of PA on the relationship between shift work with long working hours and CVD risk factors.

The prospective design is favorable, and three years is a considerable follow-up time. However, the development of e.g., cIMT is a slow process, and even though changes in cIMT were found in diabetes and coronary patients after a 12-month exercise program [56], the follow-up period may be too short to detect changes, even for subjects in a worsening state. Additionally, at the time of our investigation, many of the workers will have several years of shift work behind them, which may have, in addition to a “healthy worker effect”, an impact on further changes in some of our measures. Thus, we will strive to account for seniority in shift-work in our analyses. There is also a possibility that males and females respond differently to the circadian misalignment occurring during shift work [57], and we will strive to account for this in our analyses. However, we do not aim to investigate this issue separately in our study, and it is unlikely that we will have the optimal data set to do so, considering the male domination of workers in this occupational sector. With a follow-up period of three years, we cannot ignore the possibility of drop-outs during this part of the project. It is also of importance that participants in subgroups of PA intervention and sleep monitoring stay motivated throughout the periods of intervention and continuous measurement. Designated motivators and the involved physiotherapist from the occupational health service will be present during exercise sessions and in regular contact with all the participants. There will also be regular meetings between representatives from the participating enterprises and the project group during the three-year follow-up. This could improve compliance and limit loss to follow-up. From the sample size calculations, we regard a total sample size of 125 subjects as being large enough to show effects, even when considering a reasonable drop-out of participants.

## 4. Conclusions

This project will provide new knowledge on the relationship between one of the largest contributors to death and disability in Norway, and occupational exposure to several consecutive long shifts and night work. This knowledge has the potential to affect how work in these types of work sectors is organized, and potentially prevent or minimize detrimental health effects. It is further plausible that the study findings can be generalized to other parts of the general population.

## Figures and Tables

**Table 1:** A standard five-week shift plan for Plant A and B.

	Week 1							Week 2							Week 3							Week 4							Week 5						
Weekday	1	2	3	4	5	6	7	1	2	3	4	5	6	7	1	2	3	4	5	6	7	1	2	3	4	5	6	7	1	2	3	4	5	6	7
Plant A			E	E	E				N	N			D	D	E	E	N	N				D	D	D	D	D							N	N	N
Plant B	D	D	D	D					N	N	N	N					E	E		N	N	N				D	D	D	E	E					

D = Dayshift, E = Evening shift, N = Nightshift. Yellow color = 8 h duration of shift, Red color = 12 h duration of shift.

**Table 2:** The shift plan for Plant B during the nine months prior to plant shutdown and the standard shift plan they return to after reopening.

	Week 1							Week 2							Week 3							Week 4							Week 5						
Weekday	1	2	3	4	5	6	7	1	2	3	4	5	6	7	1	2	3	4	5	6	7	1	2	3	4	5	6	7	1	2	3	4	5	6	7
Before			N	N	N			D	D				D	D			D	D	D			N	N				N	N			N	N	N		
After	D	D	D	D					N	N	N	N					E	E		N	N	N				D	D	D	E	E					

D = Dayshift, E = Evening shift, N = Nightshift. Yellow color = 8 h duration of shift, Red color = 12 h duration of shift.

## Abbreviations

BMI	Body mass index
CD40L	CD40 ligand
cIMT	Carotis intima media thickness
CRP	C-reactive protein
CVD	Cardiovascular disease
dBP	Diastolic blood pressure
EDTA	Ethylene diamine tetra acetic acid
HbA1c	Glycosylated hemoglobin
HDL	High-density lipoprotein
HIIT	High intensity interval training
HR	Heart rate
HRV	Heart rate variablity
IL-6	Interleukin-6
LDL	Low-density lipoprotein
MCP-1	Monocyte chemoattractant protein-1
sBP	Systolic blood pressure
RPM	Revolutions per minute
TNF-*α*	Tumor necrosis factor-alpha
VO_2max_	Maximal oxygen uptake
PA	Physical activity
PAI	Personalized Activity Intelligence
PWV	Pulse wave velocity
